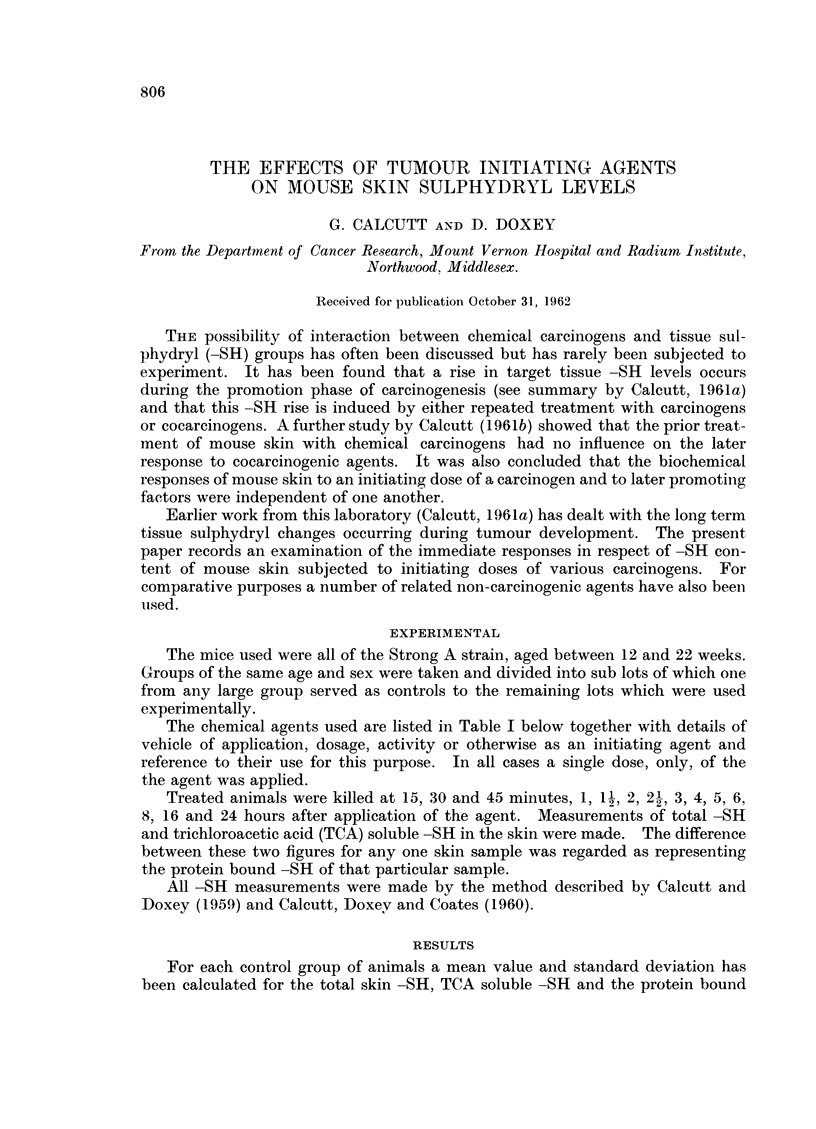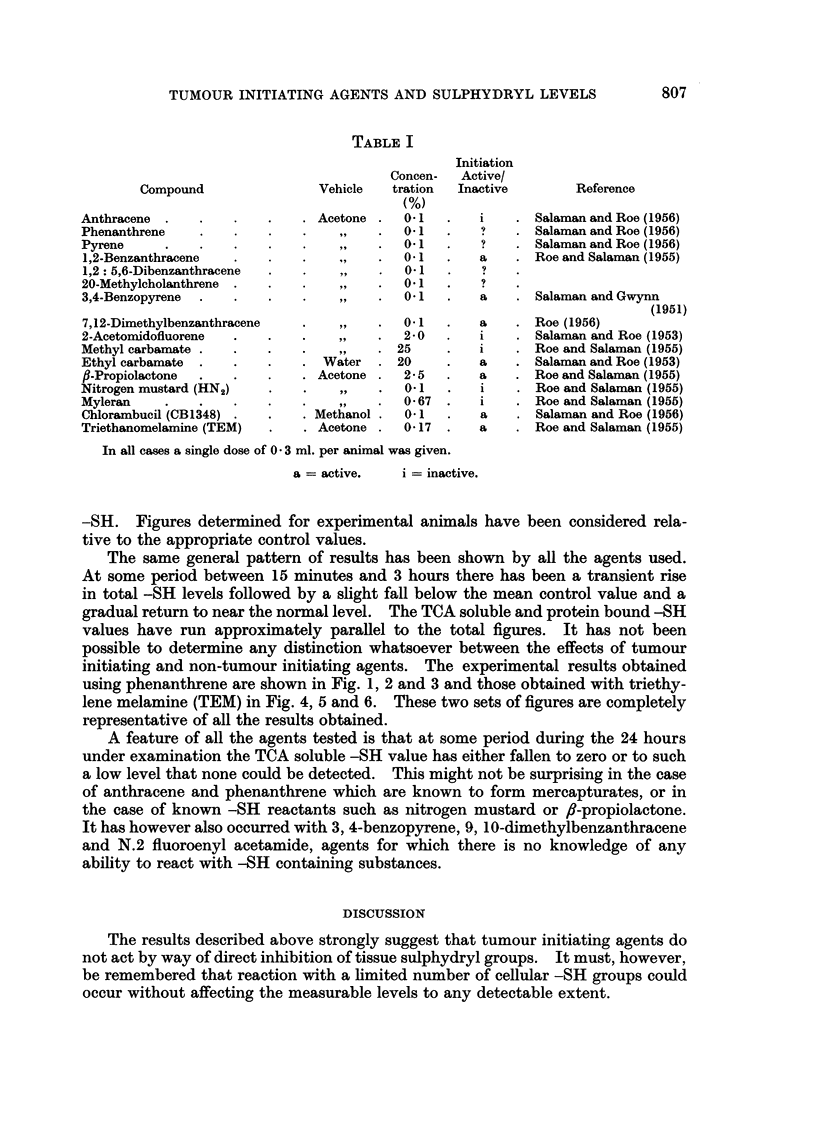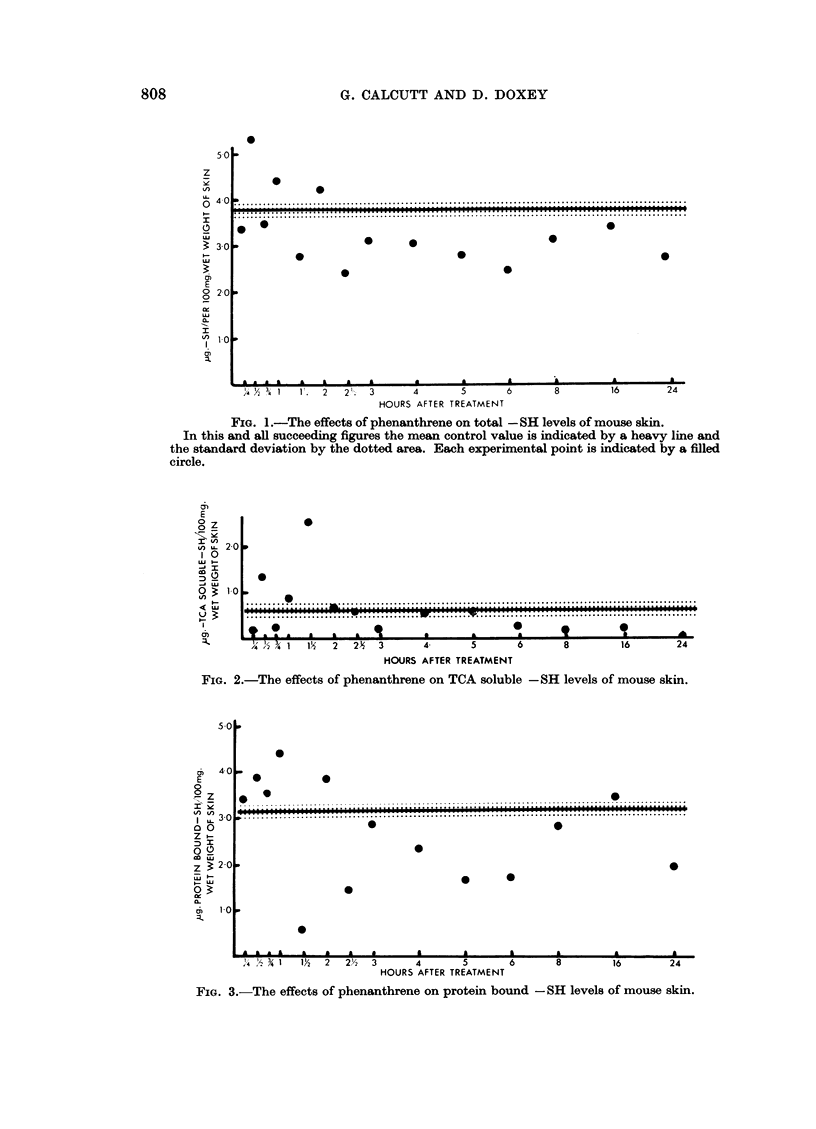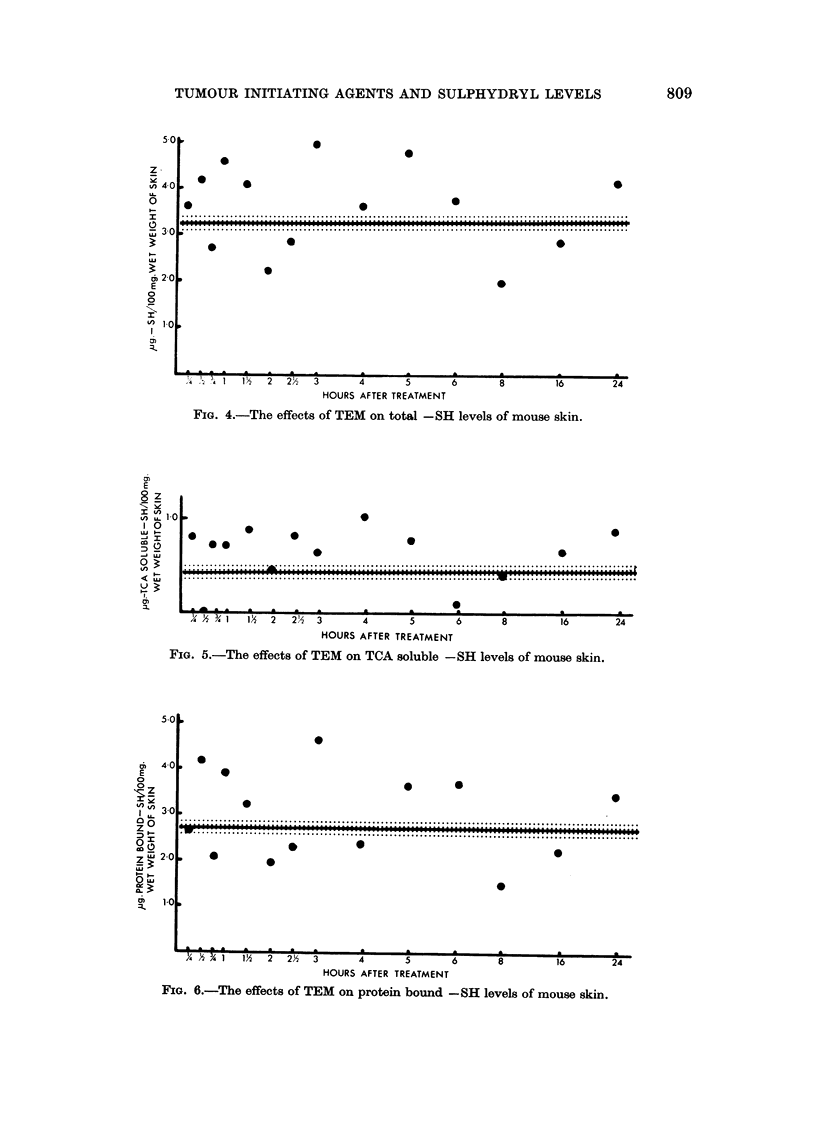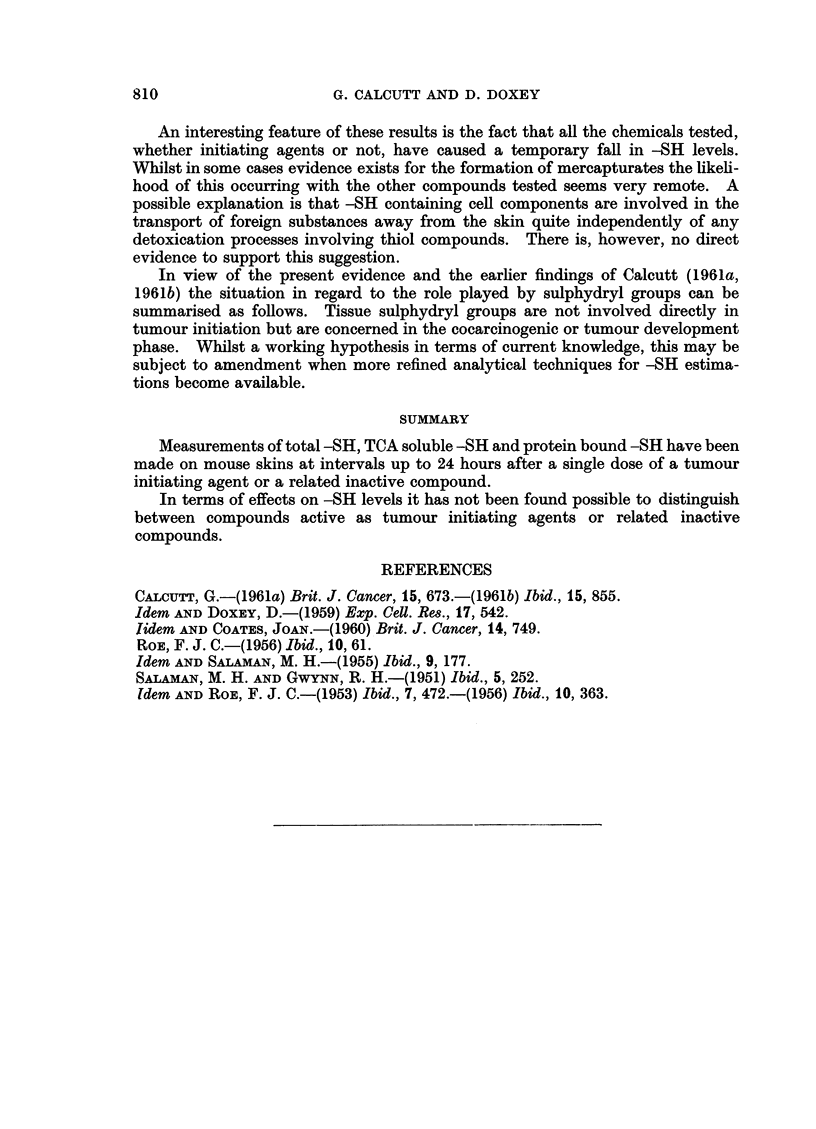# The Effects of Tumour Initiating Agents on Mouse Skin Sulphydryl Levels

**DOI:** 10.1038/bjc.1962.93

**Published:** 1962-12

**Authors:** G. Calcutt, D. Doxey


### THE EFFECTS OF TUMOUR INITIATING AGENTS ON MOUSE SKIN SULPHYDRYL LEVELS

G. CALCUTT AND D. DOXEY

*From the Department of Cancer Research, Mount Vernon Hospital and Radium Institute, Northwood, Middlesex.*

Received for publication October 31, 1962

THE possibility of interaction between chemical carcinogens and tissue sulphydryl (–SH) groups has often been discussed but has rarely been subjected to experiment. It has been found that a rise in target tissue –SH levels occurs during the promotion phase of carcinogenesis (see summary by Calcutt, 1961*a*) and that this –SH rise is induced by either repeated treatment with carcinogens or cocarcinogens. A further study by Calcutt (1961*b*) showed that the prior treatment of mouse skin with chemical carcinogens had no influence on the later response to cocarcinogenic agents. It was also concluded that the biochemical responses of mouse skin to an initiating dose of a carcinogen and to later promoting factors were independent of one another.

Earlier work from this laboratory (Calcutt, 1961*a*) has dealt with the long term tissue sulphydryl changes occurring during tumour development. The present paper records an examination of the immediate responses in respect of –SH content of mouse skin subjected to initiating doses of various carcinogens. For comparative purposes a number of related non-carcinogenic agents have also been used.

### EXPERIMENTAL

The mice used were all of the Strong A strain, aged between 12 and 22 weeks. Groups of the same age and sex were taken and divided into sub lots of which one from any large group served as controls to the remaining lots which were used experimentally.

The chemical agents used are listed in Table I below together with details of vehicle of application, dosage, activity or otherwise as an initiating agent and reference to their use for this purpose. In all cases a single dose, only, of the the agent was applied.

Treated animals were killed at 15, 30 and 45 minutes, 1, $1\frac{1}{2}$, 2, $2\frac{1}{2}$, 3, 4, 5, 6, 8, 16 and 24 hours after application of the agent. Measurements of total –SH and trichloroacetic acid (TCA) soluble –SH in the skin were made. The difference between these two figures for any one skin sample was regarded as representing the protein bound –SH of that particular sample.

All –SH measurements were made by the method described by Calcutt and Doxey (1959) and Calcutt, Doxey and Coates (1960).

### RESULTS

For each control group of animals a mean value and standard deviation has been calculated for the total skin –SH, TCA soluble –SH and the protein bound

TABLE I

| Compound | Vehicle | Concentration (%) | Initiation Active/ Inactive | Reference |
|---|---|---|---|---|
| Anthracene | Acetone | 0·1 | i | Salaman and Roe (1956) |
| Phenanthrene | ,, | 0·1 | ? | Salaman and Roe (1956) |
| Pyrene | ,, | 0·1 | ? | Salaman and Roe (1956) |
| 1,2-Benzanthracene | ,, | 0·1 | a | Roe and Salaman (1955) |
| 1,2 : 5,6-Dibenzanthracene | ,, | 0·1 | ? | |
| 20-Methylcholanthrene | ,, | 0·1 | ? | |
| 3,4-Benzopyrene | ,, | 0·1 | a | Salaman and Gwynn (1951) |
| 7,12-Dimethylbenzanthracene | ,, | 0·1 | a | Roe (1956) |
| 2-Acetomidofluorene | ,, | 2·0 | i | Salaman and Roe (1953) |
| Methyl carbamate | ,, | 25 | i | Roe and Salaman (1955) |
| Ethyl carbamate | Water | 20 | a | Salaman and Roe (1953) |
| β-Propiolactone | Acetone | 2·5 | a | Roe and Salaman (1955) |
| Nitrogen mustard ($HN_2$) | ,, | 0·1 | i | Roe and Salaman (1955) |
| Myleran | ,, | 0·67 | i | Roe and Salaman (1955) |
| Chlorambucil (CB1348) | Methanol | 0·1 | a | Salaman and Roe (1956) |
| Triethanomelamine (TEM) | Acetone | 0·17 | a | Roe and Salaman (1955) |

In all cases a single dose of 0·3 ml. per animal was given.

a = active. i = inactive.

–SH. Figures determined for experimental animals have been considered relative to the appropriate control values.

The same general pattern of results has been shown by all the agents used. At some period between 15 minutes and 3 hours there has been a transient rise in total –SH levels followed by a slight fall below the mean control value and a gradual return to near the normal level. The TCA soluble and protein bound –SH values have run approximately parallel to the total figures. It has not been possible to determine any distinction whatsoever between the effects of tumour initiating and non-tumour initiating agents. The experimental results obtained using phenanthrene are shown in Fig. 1, 2 and 3 and those obtained with triethylene melamine (TEM) in Fig. 4, 5 and 6. These two sets of figures are completely representative of all the results obtained.

A feature of all the agents tested is that at some period during the 24 hours under examination the TCA soluble –SH value has either fallen to zero or to such a low level that none could be detected. This might not be surprising in the case of anthracene and phenanthrene which are known to form mercapturates, or in the case of known –SH reactants such as nitrogen mustard or β-propiolactone. It has however also occurred with 3, 4-benzopyrene, 9, 10-dimethylbenzanthracene and N.2 fluoroenyl acetamide, agents for which there is no knowledge of any ability to react with –SH containing substances.

### DISCUSSION

The results described above strongly suggest that tumour initiating agents do not act by way of direct inhibition of tissue sulphydryl groups. It must, however, be remembered that reaction with a limited number of cellular –SH groups could occur without affecting the measurable levels to any detectable extent.

FIG. 1.—The effects of phenanthrene on total —SH levels of mouse skin.
In this and all succeeding figures the mean control value is indicated by a heavy line and the standard deviation by the dotted area. Each experimental point is indicated by a filled circle.

FIG. 2.—The effects of phenanthrene on TCA soluble —SH levels of mouse skin.

FIG. 3.—The effects of phenanthrene on protein bound —SH levels of mouse skin.

FIG. 4.—The effects of TEM on total —SH levels of mouse skin.

FIG. 5.—The effects of TEM on TCA soluble —SH levels of mouse skin.

FIG. 6.—The effects of TEM on protein bound —SH levels of mouse skin.

An interesting feature of these results is the fact that all the chemicals tested, whether initiating agents or not, have caused a temporary fall in –SH levels. Whilst in some cases evidence exists for the formation of mercapturates the likelihood of this occurring with the other compounds tested seems very remote. A possible explanation is that –SH containing cell components are involved in the transport of foreign substances away from the skin quite independently of any detoxication processes involving thiol compounds. There is, however, no direct evidence to support this suggestion.

In view of the present evidence and the earlier findings of Calcutt (1961*a*, 1961*b*) the situation in regard to the role played by sulphydryl groups can be summarised as follows. Tissue sulphydryl groups are not involved directly in tumour initiation but are concerned in the cocarcinogenic or tumour development phase. Whilst a working hypothesis in terms of current knowledge, this may be subject to amendment when more refined analytical techniques for –SH estimations become available.

SUMMARY

Measurements of total –SH, TCA soluble –SH and protein bound –SH have been made on mouse skins at intervals up to 24 hours after a single dose of a tumour initiating agent or a related inactive compound.

In terms of effects on –SH levels it has not been found possible to distinguish between compounds active as tumour initiating agents or related inactive compounds.

### REFERENCES

Calcutt, G.—(1961*a*) *Brit. J. Cancer*, **15**, 673.—(1961*b*) *Ibid.*, **15**, 855.
*Idem* and Doxey, D.—(1959) *Exp. Cell. Res.*, **17**, 542.
*Iidem* and Coates, Joan.—(1960) *Brit. J. Cancer*, **14**, 749.
Roe, F. J. C.—(1956) *Ibid.*, **10**, 61.
*Idem* and Salaman, M. H.—(1955) *Ibid.*, **9**, 177.
Salaman, M. H. and Gwynn, R. H.—(1951) *Ibid.*, **5**, 252.
*Idem* and Roe, F. J. C.—(1953) *Ibid.*, **7**, 472.—(1956) *Ibid.*, **10**, 363.